# Responsible development of clinical speech AI: Bridging the gap between clinical research and technology

**DOI:** 10.1038/s41746-024-01199-1

**Published:** 2024-08-09

**Authors:** Visar Berisha, Julie M. Liss

**Affiliations:** 1 School of Electrical Computer and Energy Engineering and College of Health Solutions, Arizona State University, Tempe, AZ USA; 2 College of Health Solutions, Arizona State University, Tempe, AZ USA

**Keywords:** Neurological disorders, Biomarkers

## Abstract

This perspective article explores the challenges and potential of using speech as a biomarker in clinical settings, particularly when constrained by the small clinical datasets typically available in such contexts. We contend that by integrating insights from speech science and clinical research, we can reduce sample complexity in clinical speech AI models with the potential to decrease timelines to translation. Most existing models are based on high-dimensional feature representations trained with limited sample sizes and often do not leverage insights from speech science and clinical research. This approach can lead to overfitting, where the models perform exceptionally well on training data but fail to generalize to new, unseen data. Additionally, without incorporating theoretical knowledge, these models may lack interpretability and robustness, making them challenging to troubleshoot or improve post-deployment. We propose a framework for organizing health conditions based on their impact on speech and promote the use of speech analytics in diverse clinical contexts beyond cross-sectional classification. For high-stakes clinical use cases, we advocate for a focus on explainable and individually-validated measures and stress the importance of rigorous validation frameworks and ethical considerations for responsible deployment. Bridging the gap between AI research and clinical speech research presents new opportunities for more efficient translation of speech-based AI tools and advancement of scientific discoveries in this interdisciplinary space, particularly if limited to small or retrospective datasets.

## Introduction

Recently, there has been a surge in interest in leveraging the acoustic properties (how it sounds) and linguistic content (what is said) of human speech as biomarkers for various health conditions. The underlying premise is that disturbances in neurological, mental, or physical health, which affect the speech production mechanism, can be discerned through alterations in speech patterns. As a result, there is a growing emphasis on developing AI models that use speech for the diagnosis, prognosis, and monitoring of conditions such as mental health^1–5^, cognitive disorders^6–10^, and motor diseases^11–15^, among others.

The development of clinical speech AI has predominantly followed a supervised learning paradigm, building on the success of data-driven approaches for consumer speech applications^16,17^. For instance, analysis of published speech-based models for dementia reveals that most models rely on high-dimensional speech and language representations^18^, either explicitly extracted or obtained from acoustic foundation models^19,20^ and language foundation models^21,22^, to predict diagnostic labels^9,23–25^; a similar trend is observed for depression^5,26^. The foundational models, initially pre-trained on data from general populations, are subsequently fine-tuned using clinical data to improve predictive accuracy for specific conditions. While data-driven classification models based on deep learning have worked well for data-rich applications like automatic speech recognition (ASR), the challenges in high-stakes clinical speech technology are distinctly different due to a lack of data availability at scale. For example, in the ASR literature, speech corpora can amount to hundreds of thousands of hours of speech samples and corresponding transcripts upon which models can be robustly trained in supervised fashion^16,17^. In contrast, currently available clinical datasets are much smaller, with the largest samples in the meta-analysis^9,24,25^ consisting of only tens to hundreds of minutes of speech or a few thousand words. This is because clinical data collection is inherently more challenging than in other speech-based applications. Clinical populations are more diverse and present with variable symptoms that must be simultaneously collected with the speech samples, ensuring proper sampling from relevant strata.

Compounding the data problem is the fact that the ground truth accuracy of diagnostic labels for different conditions where speech is impacted varies from 100% certainty to less than 50% certainty, particularly in the early stages of disease when mild symptoms are nonspecific and present similarly across many different diseases^27–34^. Retrospective data often used to train published models does not always report diagnostic label accuracy or the criteria used to arrive at a diagnosis. Collecting representative, longitudinal speech corpora with paired consensus diagnoses is time-intensive and further impedes the development of large-scale corpora, which are required for developing diagnostic models based on supervised learning. Unfortunately, supervised models built on smaller-scale corpora often exhibit over-optimistic performance in controlled environments^35^ and fail to generalize in out-of-sample deployments^36,37^. This begs the question of how we can successfully harness the power of AI to advance clinical practice and population health in the context of data availability constraints.

Here we propose that the clinical data constraints provide an opportunity for co-design of new analytics pipelines with lower sample complexity in collaboration with the clinical speech science community. The clinical speech science community has long studied the correlational and causal links between various health conditions and speech characteristics^38–42^. This research has focused on the physiological, neurological, and psychological aspects of speech production and perception, primarily through acoustic analysis of the speech signal, and linguistic analysis of spoken language. They involve interpretable and conceptually meaningful attributes of speech, often measured perceptually^43^, via functional rating scales^15^, or self-reported questionnaires^44^. Contributions from speech scientists, neuroscientists, and clinical researchers have deepened our understanding of human speech production mechanisms and their neural underpinnings, and particularly how neurodegeneration manifests as characteristic patterns of speech decline across clinical conditions^43,45^.

A co-design of a new explainable analytics pipeline can intentionally integrate scientific insights from speech science and clinical research into existing supervised models. We hypothesize that this will reduce timelines to translation, therefore providing an opportunity to grow clinical data scale through in-clinic use. As data size grows, data-driven methods with greater analytic flexibility can be used to discover new relations between speech and different clinical conditions and to develop more nuanced analytical models that can be confidently deployed for high-stakes clinical applications.

Bridging the gap between speech AI and clinical speech research leads to new opportunities in both fields. There is a clear benefit to the development of more sensitive tools for the assessment of speech for the clinical speech community. Existing instruments for assessment of speech exhibit variable within-rater and between-rater variability^46^. Developing objective proxies for these clinically-relevant constructs has the potential for increased sensitivity and reduced variability. More sensitive objective measures can also catalyze scientific discovery, enabling the identification of yet-to-be-discovered speech patterns across different clinical conditions. Conversely, effectively connecting speech AI research with clinical research enables AI developers to prioritize challenges directly aligned with clinical needs and streamline model building by leveraging domain-specific knowledge to mitigate the need for large datasets. To date, model developers have often overlooked feasibility constraints imposed by the inherent complexity of the relationship between speech production and the condition of interest. For example, recent efforts in clinical speech AI have focused on the cross-sectional classification of depression from short speech samples^5,26^. Given the well-documented variability in speech production^47^, the limitations of existing instruments for detecting depression^40^, and the heterogeneity in the manifestation of depression symptoms^48^, it is unlikely that stand-alone speech-based models will yield high-accuracy diagnostic models. Other studies have proposed using speech to predict conditions like coronary artery disease^49^ or diabetes^50^. However, to the best of our knowledge, there is no substantial literature supporting the hypothesis that speech changes are specific enough to these conditions to serve as stand-alone indicators. In working with small data sets, understanding the approximate limits of prediction is critical for resource allocation and avoiding unwarranted conclusions that could lead to premature model deployment.

**Fig. 1:**
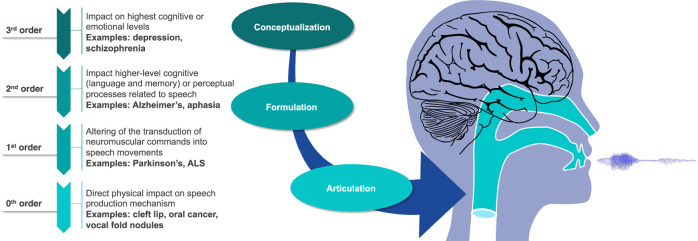
Ordering of health conditions based on their impact on speech. The production of spoken language is a complex, multi-stage process that involves precise integration of language, memory, cognition, and sensorimotor functions. The three stages are Conceptualization, Formulation, and Articulation. This figure introduces a hierarchy, or ordering, of health conditions based on how direct their impact is on the speech production mechanism.

**Fig. 2:**
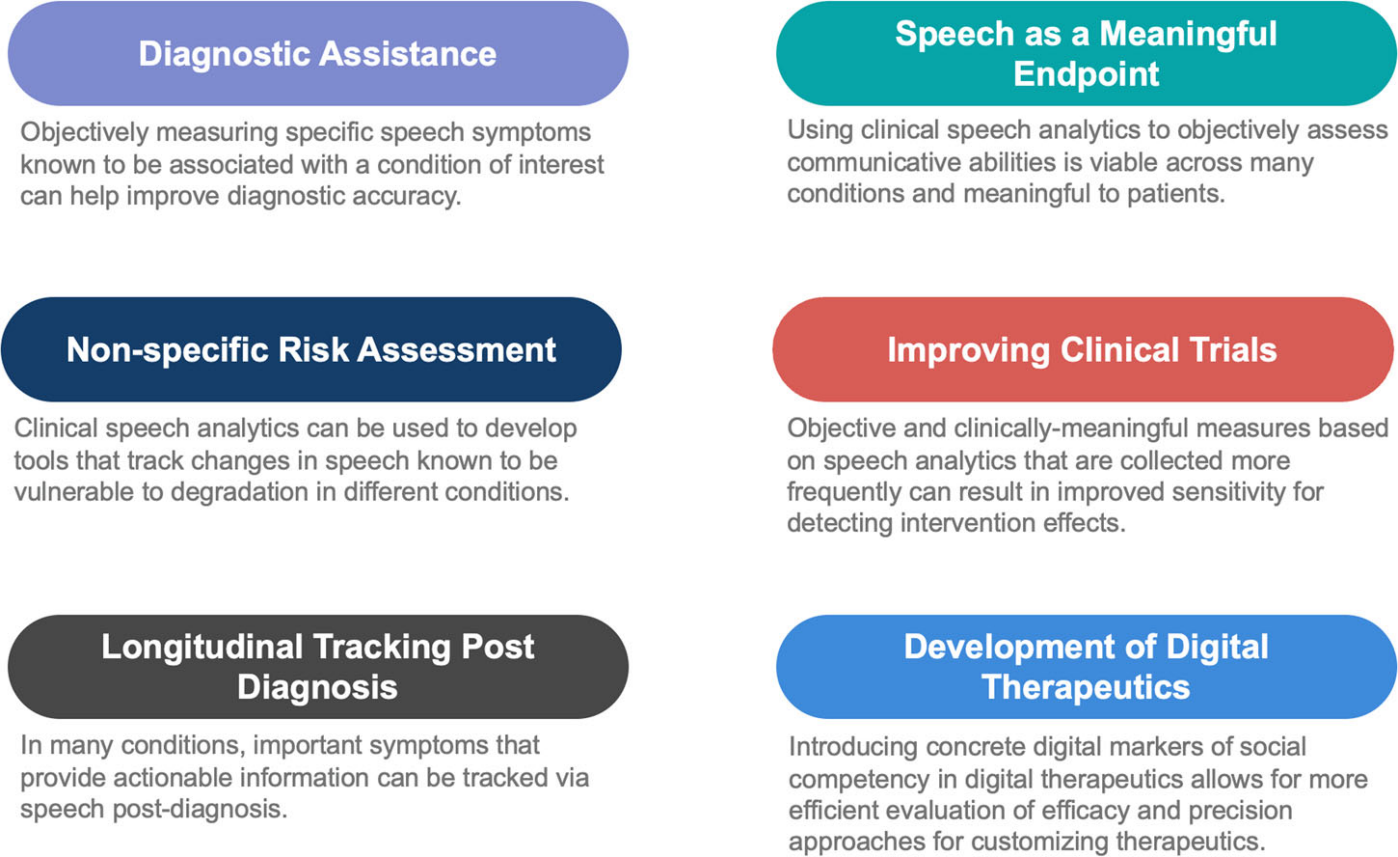
Contexts of use for clinical speech AI. A listing of different contexts of use for the development and validation of clinical tools based on speech AI.

**Fig. 3:**
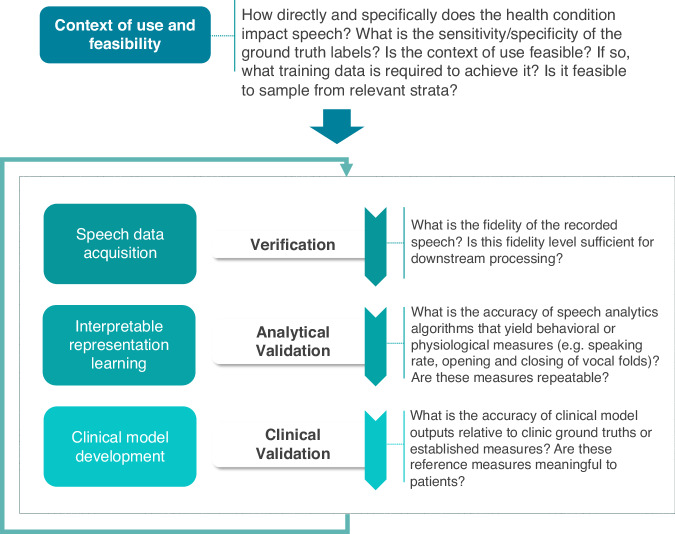
Development and validation of clinical speech AI. The development of clinical speech AI models begins with a context of use. The context of use informs downstream development and validation of resulting models. The Verification, Analytical Validation, and Clinical Validation (V3) framework has been proposed as a conceptual framework for the initial validation of biometric monitoring technologies.

**Fig. 4:**
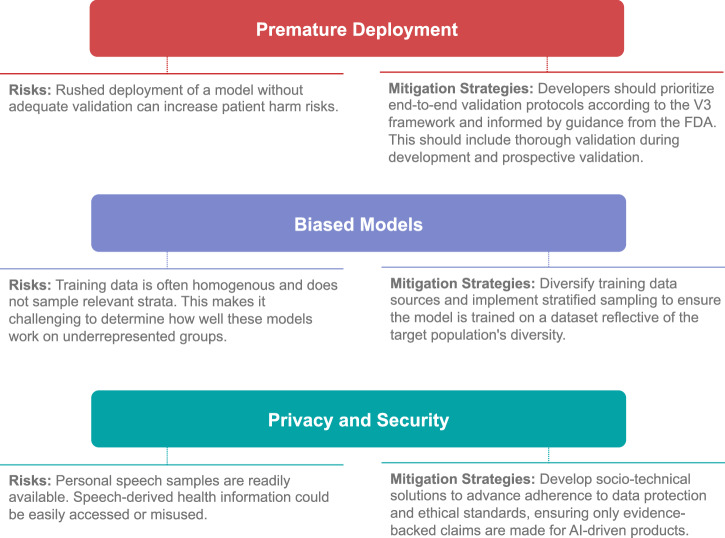
Risks and mitigation strategies for clinical speech AI. An overview of key risks and corresponding mitigation strategies for the development of clinical speech AI models.

This perspective article advocates for a stronger link between the speech AI community and clinical speech community for the development of scientifically-grounded explainable models in clinical speech analytics. We begin by presenting a new framework for organizing clinical conditions based on their impact on the speech production mechanism (see Fig. 1). We believe such a framework is important to facilitate a shared understanding of the impact of clinical conditions on speech and stimulate interdisciplinary thought and discussion. It is useful in categorizing health conditions by the complexity and uncertainty they present for speech-based clinical AI models and provides a mental model for considering the inherent limitations of speech-based classification across different conditions. It orients researchers to consider the challenges posed by limited clinical datasets during model development, and helps prevent frequent methodological errors. This has the potential to expedite progress and further foster collaboration between the speech AI community and the clinical speech community. We then explore various contexts of use for speech analytics beyond cross-sectional classification, highlighting their clinical value and the value they provide to the clinical speech research community (see Fig. 2). The discussion further examines how the selected context of use influences model development and validation, advocating for the use of lower-dimensional, individually-validated and explainable measures with potential to reduce sample size requirements (see Fig. 3). The paper concludes with a discussion on ethical, privacy, and security considerations, emphasizing the importance of rigorous validation frameworks and responsible deployment (see Fig. 4).

## The clinically-relevant information in speech

The production of spoken language is a complex, multi-stage process that involves precise integration of language, memory, cognition, and sensorimotor functions. Here we use the term ‘speech production’ to refer broadly to the culmination of these spoken language processes. There are several extant speech production models, each developed to accomplish different goals (see, for example^51–55^). Common to these models is that speech begins with a person conceptualizing an idea to be communicated, formulating the language that will convey that idea, specifying the sensorimotor patterns that will actualize the language, and then speaking^56^:

- *Conceptualization:* the speaker forms an abstract idea that they want to verbalize (Abstract idea formulation) and the intention to share through speech (Intent to speak).
- *Formulation:* the speaker selects the words that best convey their idea and sequences them in an order allowed by the language (Linguistic formulation). Then they plan the sequence of phonemes and the prosodic pattern of the speech to be produced (Morphological encoding). Next, they program a sequence of neuromuscular commands to move speech structures (Phonetic encoding).
- *Articulation:* the speaker produces words via synergistic movement of the speech production system. Respiratory muscles produce a column of air that drives the vocal folds (Phonation) to produce sound. This sound is shaped by the Articulator movements to produce speech. Two feedback loops (Acoustic feedback and Proprioceptive feedback) refine the neuromuscular commands produced during the Phonetic encoding stage over time.

Figure 1 introduces a hierarchy, or ordering, of health conditions based on how direct their impact is on the speech production mechanism. This hierarchy, motivated by initial work on speech and stress^57^, roughly aligns with the three stages of speech production and has direct consequences for building robust clinical speech models based on supervised learning.

This hierarchy compels researchers to ask and answer three critical questions prior to engaging in AI model development for a particular health condition. First, how directly and specifically does the health condition impact speech and/or language? In general, the further upstream the impact of a health condition on speech, the more indeterminate and nuanced the manifestations become, making it challenging to build supervised classification models on diagnostic labels. As we move from lower to higher-order health conditions, there are more mediating variables between the health condition and the observed speech changes, making the relationship between the two more variable and complex.

The second question the model compels researchers to ask and answer is what are the sensitivity and specificity of ground truth labels for the health condition? In general (but with notable exceptions), the objective accuracy of ground truth labels for the presence or absence of a health condition generally becomes less certain from lower to higher-order conditions, adding noise and uncertainty to any supervised classification models built upon the labels. High specificity of ground truth labels is critical for the development of models that distinguish between health conditions with overlapping speech and language symptoms. The answers to these two questions provide a critical context for predicting the utility of an eventual model prior to model building.

Finally, the hierarchy asks model developers to consider the relevant clinical speech symptoms to be considered in the model. In Table 1, we provide a more complete definition of each level in the hierarchy, a list of example conditions associated with the hierarchy, and primary speech symptoms associated with the condition. The list is not exhaustive and does not consider second and third-order impacts on speech. For example, Huntington’s disease (HD) has a first-order impact on speech causing hyperkinetic dysarthria (e.g. see Table 1). But it also has a second- and third-order impact to the extent one experiences cognitive issues and personality changes with the disease. Nevertheless, the table serves as a starting point for developing theoretically-grounded models. Directly modeling the subset of primary speech symptoms known to be impacted by the condition of interest may help reduce sample size requirements and result in smaller models that are more likely to generalize.

**Table 1:** Clinical conditions and their ordering based on the model from Fig. 1

Description	Conditions	Impact on Speech	Diagnostic Certainty
**Order 0:** Direct physical (structural or functional) effects on the speech production process. The condition or its symptoms have an immediate and evident impact on speech production mechanics. Zeroth-order health conditions directly impact the Articulation stage of speech production.	Cleft palate +/-lip or other craniofacial anomaly	Hypernasality; Articulatory distortions and substitutions^104^.	100% certainty; physically verifiable, functionally verifiable (physical examination, imaging, ultrasound); diagnostic accuracy of fetal cleft lip +/- palate is upwards of 90% accuracy^105^.
	Oral/laryngeal cancers, injury, resection	Articulatory distortions and substitutions; Phonatory abnormalities^106,107^.	High certainty; physically verifiable (physical examination, imaging).
	Vocal fold cover pathology (polyp, nodule, contact ulcers, edema)	Phonatory abnormalities^108^.	High certainty; physically verifiable (laryngoscopy).
	Aspiration	Phonatory abnormalities^109^.	High certainty when aspiration occurs during modified barium swallow videofluoroscopy.
	Structural/ functional lung conditions (e.g. COPD)	Respiratory insufficiency^110^.	High certainty; functionally verifiable (respiratory function tests).
**Order 1:** Physiological changes that alter the transduction of neuromuscular commands into speech movements. The condition causes changes in the speech apparatus or the neural pathways leading to it. Like zeroth-order health conditions, first-order conditions impact the Articulation stage of speech production, but indirectly.	Recurrent laryngeal nerve (lower motor neuron) damage during cardiac surgery	Flaccid dysarthria: breathiness from unilateral (CN X) vocal fold paralysis, diplophonia^61^.	High certainty; physically and functionally verifiable (laryngoscopy).
	Bilateral internal capsule CVAs secondary to intracranial atherosclerosis	Spastic dysarthria: strained-strangled vocal quality, slow speech, articulatory imprecision^61^.	Estimates of misdiagnosis of acute CVA range from 5% to 31%^27^.
	Parkinson’s disease	Hypokinetic dysarthria: (PD, breathy voice, imprecise articulation, monotone, reduced loudness, short rushes of speech^61^.	Estimates of misdiagnosis of early PD range from 25%–50%. Long-term presentation of chronic, hallmark symptoms plus a positive DAT reduces misdiagnosis to 10–25%^28^.
	Amyotrophic lateral sclerosis	Mixed flaccid/spastic dysarthria: slow, imprecise speech, hypernasality, vocal flutter, strained-strangled vocal quality^61^.	40% of ALS patients initially receive a false negative diagnosis; 10–15% initially receive a false positive diagnosis. Diagnosis is based on clinical examination (El Escorial Criteria) and EMG^29^.
	Huntington’s disease	Hyperkinetic dysarthria: Abnormal rate and prosody, abnormal articulatory breakdown^61^.	15% misdiagnosis rate^111^.
	Friedrich Ataxia	Ataxic dysarthria: Cerebellar degeneration, slow speech, equal and even stress, irregular breakdown^61^.	High certainty with genetic testing and symptom presentation^30^.
	Primary Progressive Apraxia of speech	Consonant and vowel distortions and substitutions; Perseverative and anticipatory errors; Metathetic syllable errors^61^.	80% misdiagnosis on the initial visit, with PAOS diagnosis taking an average of 3.4 years^31^.
**Order 2:** Conditions affecting higher-level cognitive or perceptual processes related to speech but not necessarily directly altering the physical speech apparatus. Second-order health conditions impact the Formulation stage of speech production.	CVA of left middle cerebral artery	Aphasia, either non-fluent (effortful, halting, consonant imprecision, agrammatical) or fluent (speech flows easily but lacks meaning, neologisms, jargon, auditor comprehension deficits)^112^.	Estimates of misdiagnosis of acute CVA range from 5% to 31%^27^.
	CVA of right hemisphere syndrome	Reduced affect (monotonicity, abnormal prosody); Reduced inference (concrete language, reduced recognition of humor, nuance); Articulatory, prosodic, and rate abnormalities^113^.	Estimates of misdiagnosis of acute CVA range from 5% to 31%^27^.
	Alzheimer’s disease	Anomia, empty speech, simple, general vocabulary and syntax, circumlocutions, repetitions; Articulatory, prosodic, and rate abnormalities^114^.	Estimates of AD misdiagnoses 40%; estimates of misdiagnoses of mild cognitive impairment 30% false positive^32,33^.
**Order 3:** Conditions that have their effects at the highest cognitive or emotional levels. The relationship between the condition and speech is more indirect, mediated by emotional, psychological, or high-level cognitive processes. Third-order health conditions impact the Conceptualization stage of speech production.	Psychological Conditions (Depression, Anxiety)	Articulatory, prosodic, and rate abnormalities; Abnormal amount of verbal output^4^.	Certainty of condition labels is difficult to ascertain without biological ground truth evidence. Symptom overlap among conditions further reduces the certainty of condition labels^34^.
	Psychiatric Conditions (Bipolar disorder, Schizophrenia, Psychosis)	Articulatory, prosodic, and rate abnormalities; Abnormal amount of verbal output; Language reflecting impaired Theory of Mind; Decreased coherence^4^.	Certainty of condition labels is difficult to ascertain without biological ground truth evidence. Symptom overlap among conditions further reduces the certainty of condition labels^34^.

### Ordering of health conditions based on speech impact

Zeroth-order conditions have direct, tangible effects on the speech production mechanism (including the structures of respiration, phonation, articulation, and resonance) that manifest in the acoustic signal, impacting the Articulation stage in our model in Fig. 1. This impact of the physical condition on the acoustic signal can be understood using physical models of the vocal tract and vocal folds^58^ that allow for precise characterization of the relationship between the health condition and the acoustics. As an example, benign vocal fold masses increase the mass of the epithelial cover of the vocal folds, thereby altering the stiffness ratio between the epithelial cover and the muscular body. The impact on vocal fold vibration and the resulting acoustic signal are amenable to modeling. These types of conditions are physically verifiable upon laryngoscopy, providing consistent ground truth labeling of the condition; and the direct relationship between the condition, its impact on the physical apparatus, and the voice acoustics is direct and quantifiable (although, note that differential diagnosis of vocal fold mass subtype is more difficult, see refs. ^59,60^). Thus, zeroth-order health conditions directly impact the speech apparatus anatomy and often have verifiable ground-truth labels.

First-order conditions interfere with the transduction of neuromuscular commands into movement of the articulators (e.g. dysarthria secondary to motor disorder). As with zeroth-order conditions, first-order conditions also disturb the physical speech apparatus and the Articulation stage in our model, however the cause is indirect. Injury or damage to the cortical and subcortical neural circuits and nerves impacts sensorimotor control of the speech structures by causing weakness, improper muscle tone and/or mis-scaling and incoordination of speech movements^61^. The sensorimotor control of speech movements is mediated through five neural pathways and circuits, each associated with a set of cardinal and overlapping speech symptoms: **Upper and lower motor neuron pathways; the direct and indirect basal ganglia circuits; and the cerebellar circuit**. Damage to these areas causes distinct changes in speech:

- The **lower motor neurons** (cranial and spinal nerves, originating in brainstem and spinal cord, respectively) directly innervate speech musculature. Damage to lower motor neurons results in flaccid paralysis and reduced or absent reflexes in the muscles innervated by the damaged nerves, and a flaccid dysarthria when cranial nerves are involved.
- The **upper motor neurons** originate in the motor cortex and are responsible for initiating and inhibiting activation of the lower motor neurons. Damage to upper motor neurons supplying speech musculature results in spastic paralysis and hyperreflexia, and a spastic dysarthria.
- The basal ganglia circuit is responsible for facilitating and scaling motor programs and for inhibiting involuntary movements. Damage to the **direct basal ganglia circuit** causes too little movement (hypokinesia, as in Parkinson’s disease), resulting in a hypokinetic dysarthria; while damage to the **indirect basal ganglia circuit** causes too much movement (hyperkinesia, as in Huntington’s disease), resulting in a hyperkinetic dysarthria.
- The **cerebellar circuit** is responsible for fine-tuning movements during execution. Damage to the cerebellar circuits result in incoordination, resulting in an ataxic dysarthria.

Speech symptoms are characteristic when damage occurs to any of these (or multiple) neural pathways, although there is symptom overlap and symptoms evolve in presence and severity as the disease progresses^61^. The diagnostic accuracy and test-retest reliability (within and between raters) of dysarthria speech labels from the speech signal alone (i.e., without knowledge of the underlying health condition) is known to be modest, except for expert speech-language pathologists with large and varied neurology caseloads^62^. Diagnosis of the corresponding health conditions relies on a physician’s clinical assessment and consideration of other confirmatory information beyond speech. Diagnostic accuracy is impacted by the physician’s experience and expertise, whether the symptoms presenting in the condition are textbook or unusual, and whether genetic, imaging, or other laboratory tests provide supporting or confirmatory evidence is available. For example, unilateral vocal fold paralysis is a first-order health condition with direct impact on the speech apparatus (impaired vocal fold vibration) and high-ground truth accuracy and specificity (can be visualized by laryngoscopy). In contrast, Parkinson’s disease (PD) has a diffuse impact on the speech apparatus (affecting phonation, articulation, and prosody) which is hard to distinguish from healthy speech or other similar health conditions (e.g., progressive supranuclear palsy) in early disease. The reported ground-truth accuracy of the initial clinical diagnosis ranges from 58% to 80%, calling into question clinical labels in early stage PD^28^.

Second-order conditions move away from the speech production mechanism’s structure and function and into the cognitive (i.e., memory and language) and perceptual processing domains. These conditions impact the Formulation stage of speaking and manifest as problems finding and sequencing the words to convey one’s intended message and may include deficits in speech comprehension. Alzheimer’s disease (AD) is a second-order condition that deserves particular attention because of the burgeoning efforts in the literature to develop robust supervised classification models^63^. AD disrupts the Formulation stage of speaking with word-finding problems, and the tendency to use simpler and more general semantic and syntactic structures. Natural language processing (NLP) techniques have been used to characterize these patterns and acoustic analysis has identified speech slowing with greater pausing while speaking, presumably because of decreased efficiency of cognitive processing and early sensorimotor changes^9,24,25^.

While the clinical study of speech and language in AD has consistently found evidence of such pattern changes in individuals diagnosed with probable AD, progress toward developing generalizable speech-based supervised learning clinical models for mild cognitive impairment (MCI) and AD has been relatively slow despite optimistic performance results reported in the literature^35,63^. We posit that this can be explained by answers to the first two questions that model in Fig. 1 compels researchers to consider. First, there is a lack of specificity of early speech and language symptoms to MCI and AD, given that the output is mediated by several intermediate stages and the variability associated with speech production. Mild and nonspecific speech and language symptoms will always pose a challenge for the development of clinical early detection/diagnostic speech tools until sufficient training data can result in the identification of distinct signatures (if they exist). Furthermore, given the current difficulty in accurately diagnosing MCI and AD, models based on supervised learning may be unwittingly using mislabeled training data and testing samples in their models. At present, AD is a clinical diagnosis, often preceded by a period of another clinical diagnosis of MCI. MCI is extremely difficult to diagnose with certainty, owing to variability in symptoms and their presentation over time, the overlap of speech and language symptoms with other etiologies, and the diagnostic reliance on self-report^33^. With the current absence of a definitive ground truth label for MCI or early Alzheimer’s disease, and the lack of specificity in speech changes, supervised learning models trained on small, questionably labeled data likely will continue to struggle to generalize to new data.

Third-order conditions impact the Conceptualization stage of speech production and include mental health conditions affecting mood and thought. These conditions can manifest in significant deficits and differences in speech and language, and this has been well-characterized in the literature^4^. For example, acoustic analysis can reveal rapid, pressed speech associated with mania, as well as slowed speech without prosodic variation that might accompany depression. Natural language processing can reveal and quantify disjointed and incoherent thought in the context of psychiatric disorders^64^. Despite this, the impact of these mood and thought conditions on the speech apparatus and language centers in the brain may be indirect and nonspecific relative to low-order conditions. Mental health conditions frequently cause a mixture or fluctuation of positive symptoms (e.g., hallucinations, mania) and negative symptoms (e.g., despondence, depression), which can present chronically, acutely, or intermittently. The associated speech and language patterns can be attributed to any number of other reasons (fatigue, anxiety, etc.) With regard to ground-truth accuracy and specificity, studies have shown that around half of schizophrenia diagnoses are inaccurate^65^. This problem has resulted in a push to identify objective biomarkers to distinguish schizophrenia from anxiety and other mood disorders^66,67^. This complicates the development of models for health condition detection and diagnosis; however, machine-learning models may be developed to objectively measure speech and language symptoms associated with specific symptomatology. For example, distinguishing between negative versus positive disease symptoms may be achievable with careful construction of speech elicitation tasks and normative reference data, given the central role that language plays in the definition of these symptoms^68,69^.

Across all health conditions, extraneous and comorbid factors can exert meaningful influence on speech production. For example, anxiety, depression, and fatigue, perhaps even as a consequence of an underlying illness, are known to impact the speech signal. It would not be straightforward to distinguish their influence from those of primary interest, adding complexity and uncertainty for models based on supervised learning, regardless of the health condition’s order. However, the increased variability in both data and diagnostic accuracy for many higher-order conditions makes speech-based models trained using supervised learning on small datasets vulnerable to reduced sensitivity and specificity. This is not merely a matter of augmenting the dimensionality of speech features or enlarging the dataset; it reflects the intrinsic variability in how humans generate speech. Finally, the accuracy and specificity of ground truth labels for health conditions are critical to consider in assessing the feasibility of interpretable model development. Unlike the static link between speech and the health condition, as diagnostic technologies advance and criteria evolve, the accuracy of these labels is expected to improve over time, thereby potentially enabling more robust model development.

## Defining an appropriate context of use

As mentioned before, most published clinical speech AI development studies are based on supervised learning where developers build AI models to distinguish between two classes or to predict disease severity. This approach generally presumes the same context of use for clinical speech analytics across different applications: namely, the cross-sectional detection of a specific condition or a prediction of clinical severity based on a speech sample. As we established in the foregoing discussion, this approach, when combined with limited training data, is less likely to generalize.

Nevertheless, there are a number of use cases, in which speech analytics and AI can provide more immediate value and expedite model translation. These are outlined in Fig. 2, where we explore these applications in greater depth. Focusing on these use cases will reduce timelines to translation, providing an opportunity to grow clinical data scale through in-clinic collection. With increased data size and diversity, researchers will better characterize currently-unknown fundamental limits of prediction for speech-based classification models for higher-order conditions (e.g. how well can we classify between depressed and non-depressed speech); and can bring to bear more advanced data-driven methods to problems that provide clinical value.

### Diagnostic assistance

Despite rapid advancements in biomedical diagnostics, the majority of neurodegenerative diseases are diagnosed by the presence of cardinal symptoms on clinical exams. As discussed previously and as shown in Table 1, many health conditions include changes in speech as a core symptom. For example, diagnosis of psychiatric conditions involves analysis of speech and language attributes, such as coherence, fluency, and tangentiality^70^. Likewise, many neurodegenerative diseases lead to dysarthria, and a confirmatory speech deficit pattern can be used to support their diagnoses^61^. Tools for the assessment of these speech deficit patterns in the clinical setting typically depend on the clinical judgment or on scales reported by patients themselves. There is a large body of evidence indicating that these methods exhibit variable reliability, both between different raters and within the same rater over time^46,62^. Clinical speech analytics has the potential to enhance diagnostic accuracy by providing objective measures of clinical speech characteristics that contribute to diagnosis, such as hypernasality, impaired vocal quality, and articulation issues in dysarthria; or measures of coherence and tangentiality in psychosis. These objective measures can provide utility for manual diagnosis in clinic or can be used as input into multi-modal diagnostic systems based on machine learning.

### Non-specific risk assessment tools

While differential diagnosis based on speech alone is likely not possible for many conditions, progressive and unremitting changes in certain aspects of speech within an individual can be a sign of an underlying illness or disorder^61^. Clinical speech analytics can be used to develop tools that track changes in speech along specific dimensions known to be vulnerable to degradation in different conditions. This could provide value as an early-warning indicator, particularly as the US health system moves toward home-based care and remote patient monitoring. Such a tool could be used as a non-specific risk assessment tool triggering additional tests when key speech changes reach some threshold or is supported by changes in other monitored modalities.

### Longitudinal tracking post-diagnosis

In many conditions, important symptoms can be tracked via speech post-diagnosis. For example, tracking bulbar symptom severity in ALS, as a proxy for general disease progression, can provide insights on when AAC devices should be considered or to inform end-of-life planning^71^. In Parkinson’s disease, longitudinal tracking of speech symptoms would be beneficial for drug titration^72,73^. In dementia, longitudinal tracking of symptoms measurable via speech (e.g. memory, cognitive-linguistic function) can provide valuable information regarding appropriate care and when changes need to be made.

### Speech as a clinically meaningful endpoint

Speech is our principal means of communication and social interaction. Conditions that impair speech can severely hinder a patient’s communicative abilities, thereby diminishing their overall quality of life. Current methods for assessing communication outcomes include perceptual evaluations, such as listening and rating, or self-reported questionnaires^61,69^. In contrast to the use case as a solitary diagnostic tool, employing clinical speech analytics to objectively assess communicative abilities is inherently viable across many conditions. This is due to the direct correlation between the construct (communicative ability) and the input (speech). For instance, in dysarthria, clinical speech analytics may be utilized to estimate intelligibility, the percentage of words understood by listeners, which significantly affects communicative participation^74^. In psychosis, speech analytics can facilitate the creation of objective tools for assessing social competencies; these competencies are closely tied to quality of life indicators^69^. Similarly, in dementia, a decline in social interaction can lead to isolation and depression, perhaps hastening cognitive decline^75^. A related emerging use case in Alzheimer’s disease is providing context for blood-based diagnostics. As new biomarkers with confirmatory evidence of pathophysiology emerge, there will likely be an increase in Alzheimer’s diagnoses without co-occurring clinical-behavioral features. The group of patients with AD diagnoses, but without symptoms, will require context around this diagnosis. Speech analytics will be important as measures of behavioral change that are related to quality of life.

### Improving clinical trial design

The Food and Drug Administration (FDA) prioritizes patient-relevant measures as endpoints in clinical trials. They have also identified speech and communication metrics as particularly underdeveloped for orphan diseases^76^. Objective and clinically-meaningful measures based on speech analytics that are collected more frequently can result in an improved sensitivity for detecting intervention effects. Such measures have the potential to decrease the required sample sizes for drug trials, enable more efficient enrollment, or to ascertain efficacy with greater efficiency^77^.

### Facilitating development of digital therapeutics

There has been significant recent interest in development of digital therapeutics for various neurological and mental health conditions. Several of these devices target improving the patients’ social skills or communication abilities^78^. In this evolving space, introducing concrete digital markers of social competence allows for more efficient evaluation of efficacy and precision approaches for customizing therapeutics for the patient.

## Development and validation of robust models

The context of use profoundly influences the development of clinical speech AI models, shaping their design, validation, and implementation strategies. For example, for contexts of use involving home monitoring, robustness to background noise, variability in recording conditions and usability are essential. For longitudinal monitoring, developed tools must be sensitive to subtle changes in speech characteristics relevant to the progression of the condition being monitored. This necessitates longitudinal data collection for development and validation to ensure stability and sensitivity over time. Screening tools in diverse populations require a training dataset that captures demographic variability to avoid bias. Solutions based on noisy diagnostic labels may require uncertainty modeling through Bayesian machine learning or ensemble methods that quantify prediction confidence^79^. Concurrently, techniques like label smoothing^80^ and robust loss functions^81^ can enhance model resilience under label noise.

Each context of use presents a custom development path to address the unique challenges and a parallel validation strategy that spans hardware, analytical validation, and clinical validation - see Fig. 3. The current approach focused on data-driven supervised learning on diagnostic labels limits the development and understanding of new models and makes model validation challenging. While there are many validation metrics for evaluating AI model performance, the prevalent metrics in published speech-based models primarily focus on estimating “model accuracy” (e.g. what percent of the time does the model correctly classify between Healthy and Dementia labels based on speech) using a number of methods (e.g. cross-validation, held-out test accuracy). However, accurately estimating the model accuracy of high-dimensional supervised learning models is challenging, and current methods are prone to overoptimism^35^. In addition, many supervised machine learning models are sensitive to input perturbations, which is a significant concern for speech features known for their day-to-day variability^82^. Consequently, model performance diminishes with any temporal variation in the data.

A starting point for clinical model validation is the Verification/Analytical Validation/Clinical Validation (V3) framework, a framework for validating digital biometric monitoring technologies. The original version of the framework proposes a structured approach with three evaluation levels: Verification of hardware, Analytical Validation, and Clinical Validation^83^. This framework has roots in principles of Verification and Validation for software quality product management and deployment^84^. While these existing validation systems are designed to confirm that the end system accurately measures what it purports to measure, the V3 framework adds the additional step of confirming that the clinical tools are meaningful to a defined clinical population. To that end, Verification ascertains the sensor data’s fidelity within its intended environment. Analytical validation examines the accuracy of algorithms processing sensor data to yield behavioral or physiological metrics, and clinical validation evaluates clinical model outputs with clinic ground truths or established measures known to be meaningful to patients. This includes existing clinical scales like the PHQ-9 (depression) or the UPDRS (Parkinson’s disease). In Fig. 3 we provide a high-level overview of the end-to-end development and validation process for clinical speech AI. It is important to note that the V3 is a conceptual framework that must be specifically instantiated for the validation of different clinical speech applications. While it can help guide the development of a validation plan, it does not provide one out of the box. Furthermore, this level of validation is only a starting point as the FDA suggests constant model monitoring post-deployment to ensure continued generalization^85^.

Supervised learning approaches based on uninterpretable input features and clinical diagnostic labels make adoption of the complete V3 framework challenging. Analytical validation is especially challenging as it’s difficult to ensure that learned speech representations are measuring or detecting physiological behaviors of interest. For example, in Parkinson’s disease, both the speaking rate and the rate of opening and closing of vocal folds is impacted. Uninterpretable features have unknown relationships with these behavioral and physiological parameters. As an alternative, model developers can use representations that are analytically validated relative to these constructs. This would lead to more interpretable clinical models. Validation should be approached end-to-end during the development process, with different stages (and purposes of analysis) employing different validation methods. Small-scale pilot tests may focus on parts of this framework. However, for work with deployment as a goal, ensuring generalizability and clinical utility requires validating the hardware on which the speech was collected, ensuring that intermediate representations are valid indicators of behavioral and physiological measures (e.g speaking rate, articulatory precision, language coherence), and clinical models developed using these speech measures are associated with existing clinical ground truths or scales that are meaningful to patients^86^.

Interpretable, clinically-important measures based on speech are currently missing from the literature. Clinically-relevant feature discovery and model performance evaluation in speech analytics are challenged by the high-dimensionality of speech, complex patterns, and limited datasets. Table 1 highlights several speech constructs that have been studied relative to various conditions; however, most of these constructs do not have standardized operational definitions in the clinical speech analytics literature. Instead, model developers rely on high-dimensional representations that have been developed for other purposes. For example, adopted from the ASR literature, many clinical models use representations based on mel-frequency cepstral coefficients or mel-spectra^18^; or representations learned by pre-trained foundation models^19,20^. However, these features are not interpretable, making analytical and clinical validation challenging.

Development of a clinically-tailored speech representation could significantly refine the development process, favoring smaller, individually validated, and clinically-grounded features that allow scientists to make contact with the existing literature and mitigate model overfitting and variability. This field would benefit from a concerted and synergistic effort in the speech AI community and the speech science community to operationalize and validate a measurement model for the intermediate constructs like those listed in Table 1^87^. For example, in our previous work, we made progress in this direction by developing measurement models for the assessment of hypernasality and consontant-vowel transitions and used it to evaluate cleft lip and palate and dysarthria^88,89^; several measures of volition and coherence for schizophrenia^69^; and measures of semantic relevance for dementia^10^. Individually-validated interpretable measures allow for easier alignment to different contexts of use, integration within larger multi-modal systems, and establish a more direct link to the existing clinical literature. Furthermore, they can be used as a way of explaining the operation of larger, more complex models via bottleneck constraints^90^ or they can be combined with new methods in causal machine learning for development of explainable models^91^.

Finally, clinically-interpretable representations can also play a pivotal role in integrating the patient’s perspective into the design of algorithms. The idea is that by aligning closely with the lived experiences and symptoms important to patients, these representations ensure that algorithmic outcomes resonate with the quality of life impact of health conditions. The hypothesis is that this patient-centric approach could have the added benefit of reinforcing patient trust and engagement in digital health.

## Ethical, privacy, and security considerations

The deployment and regulation of clinical speech models in healthcare present multiple challenges and risks. Prematurely launched models (without robust validation) risk delivering clinically inaccurate results and potentially causing patient harm, while biases in model training can lead to skewed performance across diverse populations. Moreover, the use of speech data for health analytics raises significant privacy and security concerns. We outline these considerations in Fig. 4 and expand on them below.

### Premature deployment of inaccurate models

A primary risk of prematurely-deployed models is that they will provide clinically inaccurate output. As discussed in previous work^35^, current strategies to validate AI models are insufficient and produce overoptimistic estimates of accuracy. Several studies have highlighted this as a more general problem in AI-based science^92,93^. However, reported accuracy metrics carry much weight when presented to the public and can lead to premature deployment. There is considerable risk that these models will fail if deployed and potentially harm patients^94^. For example, consider the Cigna StressWaves Test model, deployed after only internal evaluation and no public efficacy data. This model analyzes a user’s voices to predict their stress level and is publicly available on the Cigna Website. Independent testing of the model reveals that it has poor test-retest reliability (measured via intraclass correlation) and poor agreement with existing instruments for measuring stress^37^.

### Biased models

An additional risk of clinical speech-based models stems from the homogeneity of the data often used to train these models. Biological and socio-cultural differences contribute significantly to the variation in both the speech signal and the clinical conditions (impacting aspects from risk factors to treatment efficacy). Careful consideration of these differences in model building necessitates robust experiment design and representative stratification of data. However, a recent study demonstrates that published clinical AI models are heavily biased demographically, with 71% of the training data coming from only three states: California, Massachusetts, and New York, with 34 of the states not represented at all^95^. Similarly, analysis of clinical speech datasets indicates a significant skew towards the English language, overlooking the linguistic diversity of global populations. To accurately capture health-related speech variations, it’s essential to broaden data collection efforts to include a more representative range of the world’s native languages as health-related changes in speech can be native language-specific^96^. It becomes challenging to determine how models trained on unrepresentative data would perform when deployed for demographic groups for which they were not trained.

### Privacy and security considerations

Speech and language data is widely available and, as we continue to interact with our mobile devices, we generate an ever-growing personal footprint of our health status. Previous studies have shown that this data (speeches, social media posts, interviews) can be analyzed for health analytics^97–99^. There is a risk that similar data on an even larger scale and over longer periods of time can be accessed by technology companies to make claims about the health or emotional state of their users without their permission or by national or international adversaries to advance a potentially false narrative on the health of key figures. The risks to the privacy of this type of analysis, if used outside of academic research, is considerable, with national and international political ramifications. Internally, political adversaries can advance a potentially false narrative on the health of candidates. Internationally, geopolitical adversaries could explore this as an additional dimension of influence in elections.

There is no silver bullet to reduce these risks, however, there are several steps that can be taken as mitigation strategies. With the public availability of speech technology, building AI models has become commoditized; however, the bottleneck remains prospective validation. Thorough validation of the model based on well-accepted frames such as the V3 framework is crucial prior to deployment^83^. This validation must extend beyond initial data sets and include diverse demographic groups to mitigate biases. Moreover, developers should engage in continuous post-deployment monitoring to identify and rectify any deviations in model performance or emergent biases. Transparency in methodology and results, coupled with responsible communication to the public, can reduce the risks of misperceived model accuracy.

On the privacy front, there are emerging technical solutions to parts of this problem based on differential privacy and federated learning^100–102^; however, a complete socio-technical solution will require stringent data protection regulations and ethical guidelines to safeguard personal health information. First, it is wise to reconsider IRB review protocols in light of new technologies and publicly available data; in industry, proactive collaboration with regulatory bodies (e.g. FDA) can help establish clear guidelines. This is clear for companies focused on clinical solutions, however, the regulation of AI-based devices for technology companies, particularly those focused on wellness, is less well-defined. Recent guidance from the Federal Trade Commission (FTC) advising companies to only make evidence-backed claims about AI-driven products is a step in the right direction^103^.

## Data Availability

There is no data associated with this manuscript as it is a perspectives article centered around a theoretical framework.
